# HIP Fracture REhabilitation Program for older adults with hip fracture (HIP-REP) based on activity of daily living: a feasibility study

**DOI:** 10.1186/s12877-022-03039-x

**Published:** 2022-04-27

**Authors:** Alice Røpke, Anne-Le Morville, Trine Elleby Møller, Emma Cæcilie Guttzeit Delkus, Carsten Bogh Juhl

**Affiliations:** 1 Department of Physiotherapy and Occupational Therapy, Copenhagen University Hospital, Herlev and Gentofte, Copenhagen, Denmark; 2 Department of Sports Science and Clinical Biomechanics, University of Southern Denmark, Odense, Denmark; 3 ADULT Research Group, Department of Rehabilitation, School of Health and Welfare, Jönköping University, Jönköping, Sweden; 4 Municipality of Gentofte, Municipality of Gentofte’s Centre for Prevention and Rehabilitation, Copenhagen, Denmark; 5 Center for Training and Rehabilitation, Municipality of Lyngby-Taarbaek, Lyngby-Taarbaek, Denmark

**Keywords:** ADL, Task performance and analysis, Fractures, Hip, Rehabilitation, Pilot study

## Abstract

**Background:**

A Rehabilitation Program for older adults with hip fracture (HIP-REP) based on Activity of Daily Living has been developed. The objectives of this study were to assess the feasibility and safety of the HIP-REP program to inform a future randomized controlled trial (RCT).

**Methods:**

A feasibility study Inspired by the Complex-intervention development (Medical Research Council framework phase II) design using quantitative and qualitative research methods were conducted. Eighteen participants (above 65 years) with hip fracture were recruited from the orthopedic wards. The setting was cross sectoral including Copenhagen University Hospital, Herlev and Gentofte and rehabilitation centers in Herlev, Gentofte and Lyngby-Taarbæk municipalities. A cross-sectoral rehabilitation intervention tailored to the needs of older adults with hip fracture highlighting systematic goal setting and strategies focused on activities of daily living was conducted. Pre-defined feasibility criteria: participants recruitment and retention, duration of measuring the outcome, adherence to intervention, and adverse events, along with self-reported outcomes and an objective measurement of performance in activity of daily living. Focus groups were analyzed using a deductive manifest content analysis approach. Descriptive statistical analysis and paired *t*-tests were performed for assessing change in outcome measures.

**Results:**

Recruitment rate was 4.5/month. Outcome measures were performed but length and number of questionnaires were a burden. Thirteen out of eighteen participants completed the study three dropped out and two died. Adherence among the 13 was 100%. Focus group revealed issues regarding coordinating the intervention, ensuring procedural processes across sectors regarding recruitment of participants, and documentation in the database. Participants expressed satisfaction with the intervention and felt safe during intervention. Assessment of Motor and Process Skills showed better increase between (range 0.4 to 1.6) in ADL motor ability measures and better increase between (range 0.4 to 0.7) for process ability. No clear association between outcome improvements and intervention adherence.

**Conclusions:**

The cross-sectoral intervention based on daily activities was feasible and safe for older adults with hip fracture. A future RCT, with an improved recruitment strategy and reduced number of outcome measures will evaluate the effectiveness in improving independence and safety performance of activity of daily living.

**Trial registration:**

ClinicalTrials.gov ID: NCT03828240. Registered on January 29, 2019.

**Supplementary Information:**

The online version contains supplementary material available at 10.1186/s12877-022-03039-x.

## Introduction

Hip fracture (HF) can lead to pain, reduced ability to perform activities of daily living (ADL), and reduced quality of life [1]. Further, HF increases 1-year mortality, especially in the presence of comorbidities [2, 3]. Zidén et al. showed that multidisciplinary rehabilitation with physiotherapy and occupational therapy focusing on self-efficacy in locomotion, outdoor ambulation on independence in ADL tasks improved balance confidence, independence, and physical activity in community-dwelling older adults in the early phase after HF [4]. Furthermore, Lockwood et al. evaluated the effects of home assessment visits prior to hospital discharge for patients recovering from HF and found that home assessment visits reduced the risk of readmission to hospital, increased functional independence at six months, and may reduce the risk of falls [5]. This is in alignment with the findings by Martín-Martín et al. showing that additional structured occupational therapy involving transfer and ADL training, home environment advice, and fall prevention provided reduced emotional distress and fatigue and resulted in increased independence at one month [6]. A systematic review reported that performing ADL tasks is a challenge for many older adults with HF, and that occupational therapy interventions tend to improve occupational performance i.e., performing morning activities and using techniques for sitting, dressing and bathing [7]. As such, interventions focusing on ADL tasks seem relevant and effective for older adults with HF, but further confirmation is needed.

Therefore, an individually customized intervention program based on ADL the HIP fracture REhabilitation Program (HIP-REP), comprised of a 12-week intervention was recently developed in a collaboration between older adults with HF and healthcare professionals (HCP) [8]. The development of the HIP-REP was inspired by the Medical Research Council’s (MRC) framework for developing complex interventions [9]. However, the development stage of a program involves a number of uncertainties around the practical implementation of the program [10]. Therefore, the aim of this study was to assess the feasibility and safety of the HIP-REP intervention to inform a future randomized controlled trial (RCT). Primary outcomes included pre-defined research feasibility criteria (participant recruitment and retention, duration of measurement of the outcome, adherence to HIP-REP, and adverse events), and included evaluation using focus group interviews with occupational therapists (OTs) and older adults with HF, along with a range of secondary self-reported outcomes and an objective measurement of ADL performance.

## Methods

### Study design

This study was inspired by the second phase of the MRC framework [11] and designed as a feasibility study without a control group [12], using both quantitative and qualitative approaches. Reporting was performed according to the CONSORT statement extension to randomized pilot and feasibility trials [12].

### Participants and recruitment

Participants in this feasibility study were recruited from the orthopedic wards at Copenhagen University Hospital, Herlev and Gentofte, Denmark, by the first author (AR) and a research assistant OT between February 2019 and June 2019. Signed informed consent was obtained before enrollment. The original recruitment goal was a total of 20 participants. The inclusion criteria were men and women aged > 65 years with HFs (International Classification of Diseases 10th revision, codes S 72.0-S 72.2) [13], living at home prior to HF in Herlev, Gentofte, or Lyngby-Taarbæk municipalities, and with the ability to give informed consent. 

Exclusion criteria were severe physical impairment (e.g., not having independent walking ability pre-fracture), mental disabilities (e.g., not being able to understand instructions prior to the HF or insufficient Danish language skills), and not being expected to be discharged to own home or rehabilitation centers in the municipality. The intervention sessions were performed at the Hospital’s ward, at the rehabilitation centers, and/or in the patient’s own home. 

### Study intervention

The aim of the HIP-REP was to increase the quality of ADL performance for older adults with HFs and thereby improve their ability to live independently. The HIP-REP was tailored to the needs of older adults with HFs, in liaison with the older adults with HFs and with HCPs. The existing cross-sectoral rehabilitation pathways do not include systematic goal setting focused on ADL or home-visits after discharge from an acute hospital or rehabilitation center. As an add-on to the existing rehabilitation services, the HIP-REP therefore includes systematic goal setting and specific strategies for older adults with HF, which are individually tailored by the OTs.

### Program structure and content

The program was conducted over a total of 8 weeks as described in study the by Ropke et al [8] (Supplementary Table S1) with preliminary interviews, baseline tests, five activity-based interventions each lasting between 1 and 1.5 hours, and a phone call 10-weeks post-operation. The follow-up evaluation for this study was performed after 12 weeks.

The HIP-REP program was divided into a “two-way track” after discharge from hospital. Each track included four interventions in the municipality and was undertaken in agreement with the older participant. Track one involved older participants being discharged directly to their own homes. Track two involved participants staying at a rehabilitation center before discharge to their own homes, as shown in supplementary table S1.

The program consists of a standardized and guided manual (full version in Danish available from the authors on request) though individually tailored to the older adult. The intervention thus varies in personal activity of daily living (PADL) and instrumental activity of daily living (IADL) tasks, content and complexity, based on the older participant’s need and priorities. The HIP-REP program was guided by the Occupational Therapy Intervention Process Model (OTIPM) [14], with a focus on occupational performance for both the intervention and the evaluation, as described in the manual and as described in more detail by Ropke et al. [8]. Thirteen OTs underwent a 3-h theoretical and practical introduction, supported by a manual and detailed intervention instructions, before supervising the older adults with HFs during the intervention. 

### Outcomes

#### Evaluation of feasibility

The following five areas of feasibility were assessed: recruitment, retention, compliance/completion of the outcome measures, acceptability, and safety/adverse events (Table 1) [15]. Feasibility was registered during the baseline and follow-up measurements, supervised interventions, and the focus groups. The adverse events were registered to evaluate the safety of the program.

**Table 1:** Research progression criteria for continuing to the definitive randomized controlled trial

Outcome	Green	Amber	Red
Participant recruitment	Inclusion rate of at least 1.25 participant per week (approximately *n* = 5 per month)	(*n* < 4 after first month). If recruitment rate falls behind, reasons for exclusion will be explored after the first month to adjust eligibility criteria	No recruitment after 2 months
Completion of the outcome measures	Mean < 60 min to complete all objective outcome measures and that participants found duration acceptable	Mean < 90 min to complete all objective outcome measures	> 90 min to complete all objective outcome measures
Participant retention	Ten or more participants attend at 12-week follow-up	Only 6–9 participants attend to 12-week follow-up	Below 6 participants attend to 12-weeks follow-up
Adherence to intervention	Minimum 75% of participants adhering to at least 75% of the intervention sessions	Only 50–75% of participants adhering to 2–3 of intervention sessions	< 50% of participants adhering to intervention sessions
Adverse events	No or minor adverse events and no participants discontinuing the study	Minor or serious adverse events leading to 2 or less participants discontinuing the study	Serious adverse events leading to > 2 participants discontinuing the study

Research progression criteria were based on a traffic light of green (go), amber (amend) and red (stop) [15]. Results of these research progression criteria were evaluated by authors, who recommended whether to proceed with the definitive randomized controlled trial, and which amendments that needed to be made before proceeding

#### Baseline characteristics

Information was collected about age, gender, type of fracture, marital status, comorbidity (Charlson Index), OT and physiotherapy services, and warranted community-based assistance.

#### Primary outcomes

Recruitment procedures (i.e., eligibility rate) were evaluated by comparing the number of older adults with HF at pre-screening with participants eligible for inclusion, to identify reasons for exclusion and optimize eligibility criteria. The recruitment rate was analyzed by dividing the number of included participants by the number of months it took to include them. The duration of baseline and follow-up assessments was measured. Participant retention was evaluated by the percentage of older adults with HF who completed the 12-week follow-up. To evaluate adherence to the intervention (i.e., participation in each session with specific activities and goal setting), data were collected and managed using the secure REDCap (Research Electronic Data Capture) electronic data capture tool hosted at Herlev and Gentofte Hospital [16]. The OTs in the project all had access to the REDCap database and entered data in REDCap after each supervised intervention. Adverse events were registered in REDCap.

#### Secondary study outcome

##### Objective outcomes

To evaluate ADL ability, Assessment of Motor and Process Skills (AMPS), a standardized observation-based evaluation tool, was used to measure the observed quality of ADL task performance in terms of physical effort and fatigue, efficiency, safety, and independence [17]. The AMPS includes over 110 task descriptions, including guidelines for standardization and the varied task options. The person being tested chooses and performs two culturally relevant and familiar ADL tasks of appropriate challenge depending on the actual performance level (e.g., household management and meal preparation tasks). AMPS measures changes in motor (moving self and objects) and process (organizing and adapting actions) skills and their effect on the ability to perform complex tasks. AMPS consists of 16 motor and 20 process skill abilities that are rated on a 4-point scale. In all, 36 discrete ratings of motor and process skills were made during the observation. The rating scale is based on the following criteria for quality of performance: 4 = Competent, 3 = Questionable, 2 = Ineffective, and 1 = Severely Deficient. The ability measures are expressed in logits (log-odds probability units), and an improvement of at least 0.3 logits has been proposed as clinically meaningful and significant (i.e., indicative of improved occupational performance) [18].

##### Self-reported outcomes

The Functional Recovery Score (FRS) [19] assesses the level of physical function on an eleven-item scale comprised of three main components: basic ADL assessed by four items, instrumental activity of daily living ADL (IADL) assessed by six items, and mobility assessed by one item. Basic ADL contributes 44% of the score, IADL contributes 23%, and mobility contributes 33%. Complete independence in basic ADL, IADL, and mobility results in a score of 100%.

The European Quality of Life Scale (EQ5D-5L) [20] is a survey collecting information about health-related quality of life (HRQoL) comprising five dimensions: mobility, self-care, usual activities, pain/discomfort, and anxiety/depression. Each dimension was evaluated on 5 levels: no problems, slight, moderate, severe, and extreme problems.

The Verbal Rating Scale (VRS) is an assessment measuring the intensity of pain. The patient chooses one of the following pain ratings: none, mild, moderate, or severe [21].

The Occupational Balance Questionnaire (OBQ) [22] is an assessment measuring patients’ satisfaction with the amount and variation of their daily activities. It consists of 11 items measuring balance in daily activities on a four-step ordinal scale. For each item, the patient chooses one of the following options: disagree, partly disagree, partly agree, or agree.

The Satisfaction with Daily Occupations interview (SDO) [23] measures satisfaction with daily activities, addressing four areas of everyday occupations: work and work-related occupations, leisure occupations, domestic occupations, and self-care. The patient chooses on a scale from 1 to 7, in which 1 = Not satisfied and 7 = Very satisfied.

##### Focus groups

The older adults with HFs and the HCPs provided feedback during focus group interviews at the completion of the 12-week follow-up. One focus group included HCP participants, and another included older adults with HFs. Each focus group took place in a local area and lasted a maximum of two hours. Focus groups were led by the first author (AR) and included open-ended questions regarding the intervention and the need for change, in the following areas: 1) recruitment, e.g., the time and resources involved; 2) appropriateness of selected outcome measures, e.g., time to complete data collection or whether was it a burden for the older adults to accomplish the outcome measures; 3) retention and follow-up rates as the participants moved through the intervention, e.g., reasons for withdrawals from study; 4) adherence to the intervention, e.g., the structure, the procedure, and content of intervention; and 5) potential adverse events, e.g., how effectively adverse events were identified, documented, and reported. Both participants and OTs were asked to suggest potential improvements for the study design, structure, and procedures.

##### Sample size

The study’s aim was to evaluate the inclusion process, the intervention, the outcome measures, but not treatment effect. Though, retrospective medical record check applying trial eligibility criteria was done prior to active recruitment at the hospital to calculate how many sites was needed and how long time was needed for the recruitment [24].

##### Data analysis

Research feasibility criteria were presented with descriptive statistics. The two focus groups were recorded, transcribed, and analyzed using a deductive manifest content analysis approach, as described by Elo and Kyngäs [25]. In the preparation phase, each of the transcripts was read thoroughly several times to verify its accuracy. The organizing phase included highlighting meaning units, which were then organized and condensed using Nvivo 11 Pro [26]. An unconstrained matrix was created with different categories in the analysis procedure by the first author (AR) and overseen by the second author (AM) to ensure credibility. Continuous data was assessed for normality (histograms) and presented as mean and standard deviation (SD) when fulfilling assumptions for normality. Changes from baseline to follow-up on secondary outcomes were assessed based on normal distribution using paired *t* tests with significance level set to 5%. Stata/IC 16.1 (StataCorp. 2019) was used for the statistical analysis.

## Results

Eighteen participants were included, with a mean age of 79.4 (range 65–91), and thirteen were included in follow-up tests, with a mean age of 80.5 (range 68–91). Baseline characteristics are presented in Table 2. Except for duration of assessment, the level of acceptance was met for all research progression criteria (recruitment, participant retention, adherence, and adverse events) (Supplementary file S2). Two focus groups were carried out in September and October 2019, respectively. One group consisted of three OTs from each of the participating municipalities and one OT from the Hospital. Another focus group consisted of two older adults from different municipalities (one woman and one man). One older adult from the third municipality declined to participate in the focus group at the last minute due to illness. An inventory of physiotherapy and occupational therapy rehabilitation in addition to the HIP-REP shows that the participants were participating in group and individual physiotherapy sessions (range 6–16 h), and group and individual occupational therapy sessions (range: 1–24 h).

**Table 2:** Baseline demographic for older adults with hip fracture

Characteristic		
	Baseline (*n* = 18)	Follow-up (*n* = 13)
Age, years	79.4 (7.3) (range 65–91)	80.5 (7.2) (range 68–91)
Women *n* (%)	12 (66.6%)	10 (76.9%)
Lives alone *n* (%)	5 (27.7%)	5 (38.5%)
Use of mobility devices before admission	9 (50%)	9 (69.2%)
Type of hip fracture		
Collum femoris *n* (%)	8 (44.5%)	6 (46.6%)
Pertrochanteric *n* (%)	10 (55.5%)	7 (53.8%)
Movement restrictions *n* (%)	3 (16.7%)	3 (23.1%)
Home help before admission *n* (%)	6 (33.3%)	6 (46.6%)
Occupational therapy before admission, *n* (%)	1	1
Physiotherapy before admission, *n* (%)	4	4
Use of mobility devices before admission *n* (%)	9 (50%)	5 (38.5%)
Charlson Comorbidity Index *n* (range)	5.1 (range 3–10)	4.5 (range 3–7)
Days as inpatient *n* (range)	9.4 (range 2–42)	10.5 (range 2–42)

Data are presented as mean ± standard deviation, number (percentage), or range

### Evaluation of feasibility

#### Recruitment

**Fig. 1:**
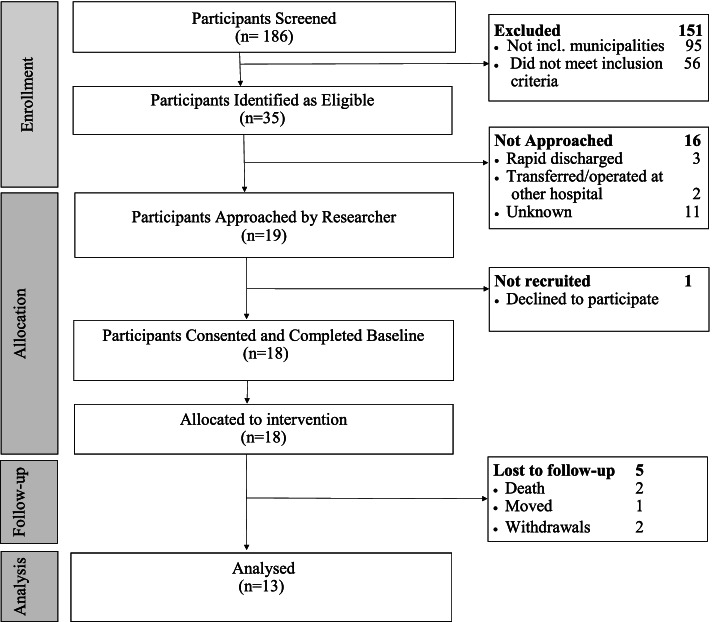
Flow diagram of participants enrolment, allocation, follow-up and analysis

Figure 1 shows that 185 potentially eligible patients admitted to Herlev and Gentofte

Hospital with HFs between February 7 and May 31, 2019 were screened. Of these patients, 151 were excluded, 95 of those due to residence in excluded municipalities and 56 due to failure to meet the study inclusion criteria for age, mental capacity, and residence at home. Thirty-five participants were identified as eligible. Due to a failure to routinely check in-patient screening lists several times daily on weekdays and to the fact that recruitment was not performed during weekends and holidays, sixteen were not approached. One declined to participate due to serious illness in the family, 18 participants (12 females) were included, and five were lost before follow-up. Over a 4-month period, this corresponds to 4.5 participants per month. An invitation to participate in the study was presented to the older adult before surgery if possible, due to the short course of hospitalization and the time needed to reflect on possible consent to the study. The purpose and the content of the intervention was easily understood by the older adults during recruitment; however, some needed to have parts of the information reviewed at the rehabilitation center or in their own homes by HCPs.

#### Completion of outcome measures

The older adults and the HCPs in the focus groups expressed that the older adults with HFs were overburdened during data collection, and that especially at baseline the participants were exhausted after filling in the questionnaires. The HCPs reported that it was difficult for participants to accomplish all the post-tests, even the 12-week post-test. Overall, the data collection plan involved a reasonable amount of time to collect data, but the length and number of questionnaires was a burden. Another issue was ensuring the right level of ADL tasks during the follow-up visit, as the planning, intervention, and completion of the primary outcome measure AMPS [18] required more time than had been allotted, depending on the OT’s knowledge of the older adult. The OTs’ suggestion was to meet and/or phone the older adult with HF just before each follow-up test to clarify the specific task chosen for the AMPS [18].

#### Retention

Thirteen out of eighteen participants completed the 12-week intervention and were included in the analysis. Two dropped out, explaining that they were independent in their ADL tasks and could get help from their spouses when necessary (one participant after the second intervention, and one who completed the intervention but declined to participate in follow-up tests). One participant completed two interventions and then moved to a summer house without completing intervention and the post-testing, and two died due to other illnesses.

#### Adherence to intervention

Thirteen participants achieved full adherence to the intervention and participated in the follow-up test. During the focus groups, the participants revealed that issues regarding successful adherence included the following:

- 1. Structure: Coordinating the add-on HIP-REP intervention with the other interventions offered at the Hospital and in the municipality such as visits by home-nurses, physiotherapy, and occupational therapy group sessions, not to mention arranging and ordering technical aids and home help presented difficulties. Ensuring that the participants received the interventions as planned according to the HIP-REP manual required time to get an overview of participants’ appointments and to coordinate appropriate placement of the HIP-REP intervention in both the older adults’ and the OTs’ calendars. It is necessary to ensure that the interventions are continued and are rescheduled if necessary, for instance, if a participant cancels an intervention due to illness or a doctor’s appointment and thereby to ensure that each participant completes the 12-week post-tests.
- 2. Procedure: When consent for participation was given, it was important to facilitate transparent cross-sectoral communication after discharge from the hospital. Furthermore, patient consent should have been noted in both electronic health records and the patient’s booklet. The project coordinators at the hospital ensured that recruitment of participants was registered to the local OTs in the municipality, who were responsible for HIP-REP intervention and follow-up tests. Another issue was the use of REDCap [16] where the HCP suggested a written introduction for new colleagues. This should include instructions on how to document and how to differentiate between the HIP-REP project and the local documentation systems. Furthermore, the HCPs discussed ideas on how to simplify the categorization of ADL tasks in REDCap to simplify registration of the intervention performed with older adults.
- 3. Content of intervention: The older adults in the focus group expressed the benefits of the presented strategies, e.g., when practicing independence in walking to the bathroom for toileting. They also mentioned having a home visit to evaluate potential environmental hazards soon after discharge as a benefit. Neither of the participants remembered a booklet with general information about HFs that was given to them during the hospital stay. An idea to solve this problem was to place each participant’s agreed-upon goals in the booklet, so the OTs in the municipality could pick up and revisit the booklet together with the older adult upon discharge to the Rehabilitation Centre or to the participant’s own home. Furthermore, the OTs suggested adding energy-saving principles for carrying out ADL tasks in the booklet. The older adults appreciated the supervised sessions and found the post-intervention follow-up phone call to be effective in addressing potential questions or concerns.

#### Adverse events

One minor event occurred during the project period: One HCP reported that when she was carrying out the HIP-REP intervention at a participant’s home, the participant fell when opening the front door for her. Fortunately, the patient was not harmed, and the incident was not considered an adverse event. None of the participants reported pain or discomfort during the intervention or testing. Participants with HFs expressed in the focus group that the supervised interventions at home were reassuring and felt like a social event. The importance of home-visits making sure of a safe home environment was highlighted in form of relevant adjustments at home or in delivery of a permanent mobility device.

### Secondary outcomes

#### Self-reported outcomes

Overall, the outcomes demonstrated few clinically significant changes following the intervention (Table 3). Self-reported outcomes showed the following: on the EQ-5D-5L, many participants reported health at the same or improved level (69%); on the VRS, most participants reported no pain (46.5%) or less pain (15.4%) at follow-up, the same level of pain as before the HF (7.7%), or some pain (23.5%) after the 3-month follow-up. No changes were seen in the SDO, OBQ, and FRS. There was no clear link between outcome improvements and intervention adherence.

**Table 3:** Secondary treatment outcomes in older adults with HF (*n* = 13)

	Baseline	Follow up	Within-group mean change (95% CI)	Range
**Observation based assessment**				
AMPS				
AMPS motor	0.00 ± 0.88	0.91 ± 0.43	0.91 (0.35, 1.46)	-0.3 to 3.1
AMPS process	0.52 ± 0.23	1.06 ± 0.45	0.52 (0.25, 0.78)	-0.3 to 1.1
**Self-reported outcome measures**				
FRS				
BADL score	15.08 ± 1.38	14.46 ± 1.85	0.66 (-0.38, 1.62)	-4 to 2
IADL score	17.23 ± 6.48	13.38 ± 5.44	3.85 (0.11, 7.59)	-14 to 4
Mobility score	3.54 ± 0.59	2.23 ± 1.01	1.31 (0.68, 1.93)	-3 to 0
Total score	34.38 ± 7.38	30.38 ± 7.06	4 (-1.33, 9.33)	-18 to 2
EQ5D-5L Health status questionnaire				
EQ-VAS, 0–100	1.66 ± 1.04	2.00 ± 1.00	-0.38 (-0.85, 0.08)	-1 to 1
Activities	1.69 ± 1.25	2.46 ± 1.13	-0.78 (-1.90, 0.37)	-4 to 3
Health	64.85 ± 22.77	65.23 ± 21.19	-0.38 (-16.52, 15.76)	-50 to 55
VRS	0.46 ± 0.78	0.38 ± 0.51	0.08 (-0.44, 0.59)	-2 to 1
OBQ				
Total score	26.38 ± 6.61	21.92 ± 7.57	4.46 (0.09, 8.83)	-15 to 12
SDO				
Number of activities	7.23 ± 2.62	6.85 ± 1.46	0.38 (-1.35, 2.12)	-8 to 2
Satisfaction with activities	76.76 ± 13.89	67.38 ± 18.85	9.38 (-4.11, 22.88)	-45 to 36
Self-rated health (1-item)	1.92 ± 1.16	1.92 ± 1.26	0 (-0.74, 0.74)	-2 to 1

Data are presented as mean ± SD

*SD* standard deviation, *CI* Confidence interval, *AMPS* Assessment of Motor and Process Score, *FRS* Functional Recovery Score, *EQ5D-5L* European Quality of Life Questionnaire, *VRS* Verbal Rating Scale, *OBQ* Occupational Balance Questionnaire, *SDO* Satisfaction of daily occupation

#### Observation-based assessment

**Fig. 2:**
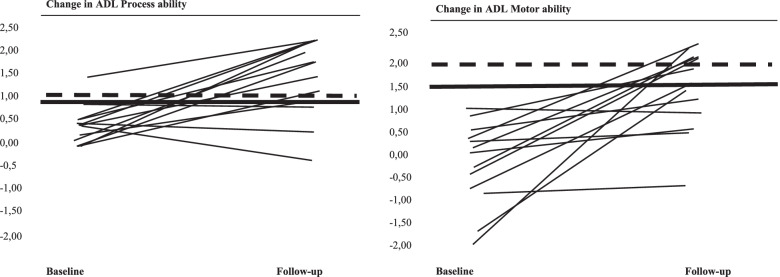
AMPS total score for every older adult with hip fracture from baseline to follow up after 12 weeks completing the HIP-REP program

In Figure 2, individual changes in AMPS measures are presented. Seven of the thirteen participants had a clinically important increase (range 0.4 to 1.6) in ADL motor ability measures, as defined in the description on the AMPS. There was a decrease in ADL motor ability measures for one participant from (1 to 0.7). Nine participants had a clinically significant increase in process ability measures (range 0.4 to 1.1). Motor ability had a mean improvement of 0.91 (95% CI -0.3 to 3.1) and process ability a mean improvement of 0.5 (95% CI -0.3 to 1.1).

## Discussion

The current study shows that the HIP-REP is feasible in terms of retention, adherence, and adverse events, while recruitment, the screening and approaching of eligible participants, the completion of outcome measures, and the duration of self-reported questionnaires need to be optimized in a future RCT.

### Recruitment

The desired number of older adults with HFs for the present study was 20, and 18 were recruited. Although the research progression criteria [15] were met for the overall recruitment, no exact information was gathered on why eleven participants identified as eligible were not invited into the project. However, there was considerable uncertainty regarding the identification of potential eligible patients during weekends and holidays from patient lists at the orthopedic ward, and new procedures taking this into account should be considered in a future RCT to ensure that the desired number of older adults with HFs is reached. Of the 19 who met the inclusion criteria and were informed at the ward with an invitation to participate in the study, only one patient declined due to family circumstances. This indicating that the older adults understand the purpose of the intervention and are motivated to participate in the development of the study. At the design stage of the intervention [8] it was important to consider that the participants in the study are frail older adults [27], and that their vulnerable situation is likely to influence the recruitment rate. A Cochrane review [28] highlights that a suitable recruitment strategy could be a face-to-face meeting between the patient and a staff member dedicated to the recruitment tasks. Furthermore, the review highlights the fact that making a specific staff member responsible might increase the chance of recruitment, and this is a strategy that will be contemplated in a future RCT. Regarding the oral and written information, the strategy of being in direct contact with the eligible older adult was deliberately chosen during the design phase of the intervention and was confirmed as a good measure by the HCPs at a focus group meeting. Issues regarding how many sessions and how much time the intervention was expected to take, including face-to-face sessions either at the rehabilitation center or in their own homes (and excluding transportation time), were highlighted and clarified, as recommended by Treweek [29] and Prescott et al [30].

### Retention

Thirteen older adults completed the 8 weeks of intervention. Two participants died because of other underlying diseases, which is normal considering the population. Three dropped out during the study, two because they found themselves sufficiently independent in performing ADL tasks and another due to relocation outside of the municipality. The reasons for drop-out are difficult to predict and prevent, and therefore it is recommended that a future RCT takes possible drop-outs into account when establishing the desired sample size. A friendly and safe atmosphere is essential for preventing drop-outs [31]. Based on feedback from focus groups, such an atmosphere was achieved in this study, and thus was not a reason for the dropouts.

During the data collection, the HCPs questioned whether to follow the manual’s planned sessions for specific weeks. Some participants regained their pre–hip-fracture independence in ADL before the end of the 12 weeks, and for others it took longer because adjacent complications occurred. Furthermore, vacations, appointments with doctors, and other obligations could cause a session to be moved to another date. However, it was agreed that the older adults should receive their planned sessions as close as possible to the timeline described in the manual.

### Completion of outcome measures

The included participants were all living in their own homes before the HF incidence, five living alone and thirteen with a partner or family member. All thirteen remaining participants provided outcome data at 12 weeks post operation, and the results showed that participants had improved in ADL motor and ADL process abilities. The mean changes in the participants’ ADL ability measures showed that they performed ADL tasks with less effort, increased efficiency, less safety risk, and less need of assistance. This suggests that AMPS is sensitive to patients with HF and can be used in a definitive RCT. However, feedback from HCPs highlighted that selecting tasks with the appropriate degree of difficulty from those suggested in the AMPS manual may be a challenge, especially for men, either because they may never have performed the suggested tasks or because their spouses usually perform them. Comparing pre- and post-intervention AMPS measures provides the objective evidence needed to determine if an improvement occurred. Due to the individual calibration of AMPS testers, the OT does not have to observe the older adults performing the same or even similar AMPS tasks, and different OTs can administer the AMPS observations from baseline to follow-up. However, the study also showed the importance of the baseline AMPS measurements, when the OT gathers information about the older adult’s current performance, context and to prepare the types of ADL tasks that possibly will be appropriately challenging and realistic to observe when reevaluating the AMPS follow-up [17].

To clarify the Specific Task Contract [18], the OT must determine if the older adult is familiar with the Environment, ensure that appropriate tools and materials are available, and select two appropriately challenging ADL tasks to observe. The selection of AMPS tasks is presented by the OT AMPS tester, although it can be a challenge to select tasks that motivate the older adult, especially older men with HFs, as many of the tasks are based on meal preparation and are not necessarily ADL tasks they usually perform. This preparation takes time, which suggests that an additional hour is needed for the follow-up test visit when planning for a future RCT.

Some improvements were also observed in the EQ5D-5L, but none in the VRS, FRS, SDO, or OBQ. The baseline of these latter three outcome measures was based on the participants’ pre-fracture function. The result of the FRS at the 12-week follow-up endpoint showed that most participants did not regain their pre-fracture function levels. This might indicate that a 6- or 12-month follow-up measurement would be more appropriate. According to a review by Dyer et al. [32], 34–59% of all HF patients regain their basic ADL after three months and 42–71% after six months. Another study by Moerman et al. reports that less than one-third of HF patients return to their pre-fracture level of instrumental ADL, and the three-month follow-up was not sufficient to see a change in functional recovery, especially in IADL [33].

The duration of baseline and follow-up assessments was beyond the pre-defined acceptable level of < 60 min. Both the HCPs and the older adults found the assessments too time-consuming and too tiring for the older adults. The participants pointed out that there were some repeated questions in the assessments, such as self-rated health questions and questions regarding ADL experienced in the SDO and OBQ assessments. The SDO assessment was especially exhausting, with many questions and ratings.

### Adherence to intervention

Considering the target group of older, vulnerable adults, studies highlight the importance of not overburdening participants with excessive physical exercise and information [34]. The information presented when participants are invited to take part in the study should be beneficial, short, and to the point, otherwise they will turn down the offer. During the feasibility study, the older adults quickly understood the purpose of the study and expressed relief when told that sessions would take place where they were situated, either at a rehabilitation center or at home.

Possible barriers for successful adherence, especially regarding performing ADL tasks during the hospital stay, include the time and the surroundings needed to perform the intervention [35, 36]. Due to the need to incorporate time to consider consent, collect baseline measurements while at the hospital, attend to prioritized examinations such as x-rays, and participate in mobilization sessions with the physiotherapist, little time was left to plan and perform ADL tasks at the ward. To increase adherence, adjustments to the HIP-REP program may be beneficial. In the municipality, the OT-supervised interventions were performed at the rehabilitation centre or at the older adult’s own home. Eliminating travel and travel-related expenses for the older adult was contemplated in the design phase as a way to minimize adherence barriers, as Treweek [29] suggests. The shared responsibilities between the OTs in the municipalities and rehabilitations centers need to be coordinated to a greater extent for interventions with the older adults in the HIP-REP program. The OTs experienced issues with their shifting roles between being in a rehabilitation center and being in a home setting, due to the differences in physical environment and different degrees of access to technical aids.

When implementing new interventions, HCPs can be challenged in learning new procedures and skills [37]. Although the HIP-REP program is described in the intervention manual, further written information for the provider of an intervention should be included in the introduction, along with ongoing support from research staff to ensure intervention adherence [38]. Furthermore, there was a need to simplify the OTs’ registration of interventions in the REDCap system to reduce workload and avoid confusion, e.g., to categorize ADL tasks in domains in the REDCap system to streamline registration of the intervention carried out.

### Adverse events

Only one minor event occurred in connection with a home visit, and the participants expressed that they felt safe during the intervention and that the follow-up phone call improved their feeling of safety in everyday life. However, they still found it necessary to have enhanced focus on barriers in the home and adjustments to improve accessibility in the home environment, such as a grab bar at the balcony or a mobility device for outdoor activities.

### Strengths and limitations

Our study has some limitations, as the effectiveness of the HIP-REP program cannot be evaluated, due to the lack of a control group receiving usual care. This was a deliberate decision, since the intention was to evaluate feasibility rather than effectiveness. Regarding statistics normal distribution would require more participants to be valid and should therefore be interpreted with caution. However, due the sensitivity of our main outcome, the AMPS test makes the number of participants sufficient [17]**.**

When conducting multicenter trials, the feasibility of different elements in the study can act as an opportunity to test whether collaboration can be developed, and processes can run across sectors and multiple centers [39]. To further enhance external validity consecutive sampling was used in the selection of the participants, including 18 from 35 eligible participants. This ensured controlling sampling bias because all available subjects were included.

Furthermore, allowing the calculation of a response rate [40]. Comparing the 18 included participants’ age, gender and fracture type with data from the Danish Multidisciplinary Hip Fracture Registry [41], shows similarity in age, gender and mostly medial and pertrochanteric femur fracture. Another strength of our study was the use of specifically trained and calibrated AMPS raters to address interventions aiming at improving ADL ability. All were experienced in planning, implementing, and evaluating interventions focusing on ADL ability.

Engaging the OTs early in determining the feasibility of the research and the complex intervention increased the chances that the research will inform a change in future practice [42]. Collecting both quantitative (psychometric properties) and qualitative (focus groups) data ensured a deeper understanding of the participants’ experience with the intervention [43, 44]. Hence, to evaluate to what extent the data collection procedures and outcome measures were feasible and appropriate for the study, the focus group method was chosen to collect qualitative data from the older adults and the HCPs. However, it was difficult to recruit participants, as the travel distance to the meetings and the duration of the interviews made the focus group process time consuming for the HCPs and exhausting for the older adults. A larger number of the older adults and HCPs may have further qualified the feedback on the HIP-REP intervention. However, the contribution using patient and HCP inputs to process evaluation cross-sectoral from the involved older adults and HCP’s in focus groups gave invaluable information to the study.

## Conclusions

In conclusion, the HIP-REP program is feasible and safe for older adults with HFs. Clarifying the worksheets for the user manual and REDCap will further optimize the use of the written instructions for the HCPs. During an RCT, we recommend that the recruitment procedure is followed more carefully to increase the number of participants included. Furthermore, it is necessary to decrease the number of outcome measures, in order to reduce the burden and duration of the test. However, even though the study was able to detect clinically important changes in ADL ability and to a certain extent in self-reported health variables, we can only consider the potential effects of the intervention after a full-scale evaluation of the intervention in a clinical trial has been completed.

## Supplementary Information

**Additional file 1: S1.** HIP-REP program for older adults with hip fracture from first post-operative day to Week 12 including five interventions based on Occupational performance: One intervention during hospital stay and four at the rehabilitation center and/or at home. Home visits must be carried out in both tracks 1 and 2.

**Additional file 2:** **S2.** Primary outcomes in research progression criteria to inform the definitive randomized controlled trial.

## Data Availability

Data supporting the findings are available on request. Please contact the corresponding author, Alice Ropke (aliceropke@me.com) for data access.

## Abbreviations

HIP-REP: HIP fracture Rehabilitation program
RCT: Randomized controlled trial
ADL: Activity of daily living
HF: Hip fracture
HCP: Healthcare professional
MRC: Medical Research Council;
OT: Occupational therapist
PADL: Personal activity of daily living
IADL: Instrumental activity of daily living
OTIPM: Occupational Therapy Intervention Process Model
REDCap: Research Electronic Data Capture
AMPS: Assessment of Motor and Process Skills
FRS: Functional Recovery Score
EQ5D-5L: European Quality of Life Scale
HRQoL: Health-related quality of life
VRS: Verbal Rating Scale
OBQ: Occupational Balance Questionnaire
SDO: Satisfaction with Daily Occupations interview

## References

[1] Bentler SE, Liu L, Obrizan M, Cook EA, Wright KB, Geweke JF (2009). The aftermath of hip fracture: discharge placement, functional status change, and mortality. Am J Epidemiol.

[2] Berggren M, Stenvall M, Englund U, Olofsson B, Gustafson Y (2016). Co-morbidities, complications and causes of death among people with femoral neck fracture - a three-year follow-up study. BMC Geriatr.

[3] de Luise C, Brimacombe M, Pedersen L, Sørensen HT (2008). Comorbidity and mortality following hip fracture: a population-based cohort study. Aging Clin Exp Res.

[4] Zidén L, Frändin K, Kreuter M (2008). Home rehabilitation after hip fracture. A randomized controlled study on balance confidence, physical function and everyday activities. Clin Rehabil.

[5] Lockwood KJ, Harding KE, Boyd JN, Taylor NF (2019). Predischarge home visits after hip fracture: a randomized controlled trial. Clin Rehabil.

[6] Martín-Martín LM, Valenza-Demet G, Jiménez-Moleón JJ, Cabrera-Martos I, Revelles-Moyano FJ, Valenza MC (2014). Effect of occupational therapy on functional and emotional outcomes after hip fracture treatment: a randomized controlled trial. Clin Rehabil.

[7] Lee SY, Jung SH, Lee SU, Ha YC, Lim JY (2019). Is Occupational Therapy After Hip Fracture Surgery Effective in Improving Function?: A Systematic Review and Meta-Analysis of Randomized Controlled Studies. Am J Phys Med Rehabil.

[8] Ropke A, Lund K, Thrane C, Juhl C, Morville AL (2021). Developing an individualised cross-sectoral programme based on activities of daily living to support rehabilitation of older adults with hip fracture: a qualitative study. BMJ Open..

[9] Craig P, Dieppe P, Macintyre S, Michie S, Nazareth I, Petticrew M (2008). Developing and evaluating complex interventions: the new Medical Research Council guidance. BMJ..

[10] Richards D, D Richards IH (2015). Complex Interventions in Health An overview of research methods. The Complex Interventions Framework.

[11] Craig P, Dieppe P, Macintyre S, Michie S, Nazareth I, Petticrew M (2013). Developing and evaluating complex interventions: the new Medical Research Council guidance. Int J Nurs Stud.

[12] Eldridge SM, Chan CL, Campbell MJ, Bond CM, Hopewell S, Thabane L (2016). CONSORT 2010 statement: extension to randomised pilot and feasibility trials. BMJ.

[13] World Health Organization. International Classification of Diseases (ICD). WHO. Oct 31, 2021: Available at: https://icd.who.int/browse10/2019/en#/S72.0

[14] Fisher AG (2013). OTIPM.

[15] Avery KNL, Williamson PR, Gamble C, O’Connell Francischetto E, Metcalfe C, Davidson P (2017). Informing efficient randomised controlled trials: exploration of challenges in developing progression criteria for internal pilot studies. BMJ Open..

[16] Harris PA, Taylor R, Thielke R, Payne J, Gonzalez N, Conde JG (2009). Research electronic data capture (REDCap)—A metadata-driven methodology and workflow process for providing translational research informatics support. J Biomed Inform.

[17] Fisher AG, Jones KB (2011). Assessment of Motor and Process Skills: User manual.

[18] Fisher AG, Jones KB (2010). Assessment of Motor and Process Skills: Skills, Development, standardization and administration: Manual.

[19] Danske Fysioterapeuter [Danish Physiotherapists]. Functional Recovery Score: Geriatric Hip fracture Research Group; 2018. Dec 20, 2021: Available: http://fysio.dk/fafo/Maleredskaber/

[20] Tidermark J, Bergström G, Svensson O, Törnkvist H, Ponzer S (2003). Responsiveness of the EuroQol (EQ 5-D) and the SF-36 in elderly patients with displaced femoral neck fractures. Qual Life Res.

[21] Bech RD, Lauritsen J, Ovesen O, Overgaard S (2015). The Verbal Rating Scale Is Reliable for Assessment of Postoperative Pain in Hip Fracture Patients. Pain Res Treat.

[22] Wagman P, Hakansson C (2014). Introducing the Occupational Balance Questionnaire (OBQ). Scand J Occup Ther.

[23] Eklund M, Gunnarsson AB (2007). Satisfaction with Daily Occupations: Construct validity and test–retest reliability of a screening tool for people with mental health disorders. Aust Occup Ther J.

[24] Treweek S, Richards DHI (2015). Addressing issues in recruitment and retension using feasibility and pilot trials. Complex Interventions in Health An overview of research methods.

[25] Elo S, Kyngäs H (2008). The qualitative content analysis process. J Adv Nurs.

[26] QSR International Pty Ltd. (2017) NVivo (Version 11), https://www.qsrinternational.com/nvivo-qualitative-data-analysis-software/home

[27] Wilson H (2014). Multi-disciplinary care of the patient with acute hip fracture: How to optimise the care for the elderly, traumatised patient at and around the time of the fracture to ensure the best short-term outcome as a foundation for the best long-term outcome. Best Pract Res Clin Rheumatol.

[28] Preston NJ, Farquhar MC, Walshe CE, Stevinson C, Ewing G, Calman LA (2016). Strategies designed to help healthcare professionals to recruit participants to research studies. Cochrane Database Syst Rev.

[29] Treweek S, Richards DHI (2015). Addresseing issues in recruitment and retension using feasibility and pilot trials. Complex Interventions in Health An overview of research methods.

[30] Prescott RJ, Counsell CE, Gillespie WJ, Grant AM, Russell IT, Kiauka S (1999). Factors that limit the quality, number and progress of randomised controlled trials. Health technol assess.

[31] Sarah B, David MM, Michelle GP, Karen M, Susan R, Kathye EL (2006). Lessons Learned in Participant Recruitment and Retention: The EXCITE Trial. Phys Ther.

[32] Dyer SM, Crotty M, Fairhall N, Magaziner J, Beaupre LA, Cameron  ID (2016). A critical review of the long-term disability outcomes following hip fracture. BMC Geriatr.

[33] Moerman S, Mathijssen NMC, Tuinebreijer WE, Nelissen RGHH, Vochteloo AJH (2018). Less than one-third of hip fracture patients return to their prefracture level of instrumental activities of daily living in a prospective cohort study of 480 patients. Geriatri Gerontol Int.

[34] Treweek S, Pitkethly M, Cook J, Fraser C, Mitchell E, Sullivan F (2018). Strategies to improve recruitment to randomised trials. Cochrane Database Syst Rev.

[35] Ross S, Grant A, Counsell C, Gillespie W, Russell I, Prescott R (1999). Barriers to Participation in Randomised Controlled Trials: A Systematic Review. J Clin Epidemiol.

[36] Walsh DM, Howe TE, Johnson MI, Moran F, Sluka KA (2015). Transcutaneous electrical nerve stimulation for acute pain. Cochrane Database Syst Rev.

[37] O’Cathain A, Croot L, Duncan E, Rousseau N, Sworn K, Turner KM (2019). Guidance on how to develop complex interventions to improve health and healthcare. BMJ Open..

[38] Proctor EK, Powell BJ, McMillen JC (2013). Implementation strategies: recommendations for specifying and reporting. Implement Sci..

[39] Avery PP, Baker RP, Walton MJ, Rooker JC, Squires B, Gargan MF (2011). Total hip replacement and hemiarthroplasty in mobile, independent patients with a displaced intracapsular fracture of the femoral neck: A seven-to ten-year follow-up Report of a Prospective randomised controlled trial. J Bone Joint Surg, Br.

[40] Polit D, Bech C. Essentials of nursing of nursing practice. Philadelphia: Wolters Kluver; 2017.

[41] Danish Interdisciplinary Register for Hip Thigh Bone Fracture [Dansk tværfagligt register for hoftenære lårbensbrud]; 2019. Oct 29, 2021: Available from: https://www.sundhed.dk/content/cms/62/4662_hofte_lprrapport_2019_endelig_off.pdf).

[42] Giangregorio LM, Thabane L, Richards AH IR (2015). Complex Interventions in Health.. Pilot studies and feasibility studies for complex interventions.

[43] Orsmond GI, Cohn ES (2015). The Distinctive Features of a Feasibility Study: Objectives and Guiding Questions. OTJR.

[44] Feeley N, Cosette S, Richards DHI (2015). Testing the waters. Complex Interventions in Health.

