# Supplementary material for: HIP Fracture REhabilitation Program for older adults with hip fracture (HIP-REP) based on activity of daily living: a feasibility study
Source: BMC Geriatr. 2022 Apr 27;22:370. doi: 10.1186/s12877-022-03039-x (PMC9044869; doi:10.1186/s12877-022-03039-x)
Supplement: Supplementary file 1 — Additional file 1: S1. HIP-REP program for older adults with hip fracture from first post-operative day to Week 12 including five interventions based on Occupational performance: One intervention during hospital stay and four at the rehabilitation center and/or at home. Home visits must be carried out in both tracks 1 and 2. [file 12877_2022_3039_MOESM1_ESM.docx]

**S1** HIP-REP program for older adults with hip fracture from first post-operative day to Week 12 including five interventions based

on Occupational performance: One intervention during hospital stay and four at the rehabilitation center and/or at home. Home visits

must be carried out in both tracks 1 and 2

| The Progress at the hospital | | | | | | |
| --- | --- | --- | --- | --- | --- | --- |
| Day Post  Operative | **Session** | **Intervention** | | **Activities** | | |
| Day 1 | 1st | Inform and identify | | Welcome to the ward | |  |
| Day 1-2 | 2nd | Inform and identify | | Initial interview | |  |
|  |  |  |  | Interview – prioritize two ADL tasks for AMPS | |  |
| Day 3 | 3rd | Inform and identify  Objectives, planning and implementation | | Observation: AMPS, as well as clarifying and interpreting cause and discussing objectives | |  |
|  |  |  |  | Hip fracture information; operation, restriction movements handed out | |  |
| Day 3-4 | 4th | First ADL intervention  Inform and identify, goalsetting, plan, engage and assess results | | PADL and IADL tasks at the ward prioritized by older adult | |  |
| Day 4-5 | 5th | Evaluate and end course | | Clarify and order assistive devices | |  |
| The Progress in the municipality | | | | | | |
|  | | **Track 1**  **Discharge from Hospital to in-patient Rehabilitation Centre to own dwelling** | | **Track 2**  **Direct discharge from hospital to own dwelling** | | |
| Week post- operative | **Session** | **Intervention** | **Activities** | **Intervention** | **Activities** | |
| Week 2 | 6th | Second ADL intervention  Inform and identify, goalsetting, plan, engage and assess results | Welcome/ Initial conversation | Second ADL intervention at home  **Weekday 1-3 after discharge**  Inform and identify, goalsetting, plan, engage and assess results | The accessibility of the housing is reviewed |  |
|  |  |  | PADL and IADL tasks as the older adult has prioritized |  | Review of ADL tasks in own residence |  |
| Week 3 | 7th | third ADL intervention at home if possible.  Inform and identify, goalsetting, plan, engage and assess results | PADL and IADL tasks as the older adult has prioritized | Third ADL intervention at home  Inform and identify, goalsetting, plan, engage and assess results | PADL and IADL tasks as the older adult has prioritized |  |
| Week 5 | 8th | Fourth ADL intervention  **Home visit in connection with discharge from Rehabilitation Centre**  Inform and identify, goalsetting, plan, engage and assess results | The accessibility of the housing is reviewed | Fourth ADL intervention at home  Inform and identify, goalsetting, plan, engage and assess results | PADL and IADL tasks as the older adult has prioritized |  |
|  |  |  | Review of ADL tasks in own residence |  |  |  |
| Week 8 | 9th | Fifth ADL intervention  **In own home**  Inform and identify, goalsetting, plan, engage, assess results, and end course | PADL and IADL tasks as the older adult has prioritized | Fifth ADL intervention at home  Inform and identify, goalsetting, plan, engage, assess results, and end course | PADL and IADL tasks as the older adult has prioritized |  |
|  |  |  | End course and evaluate |  | End course and evaluate |  |
| Week 10 | Phone-call | Assess results and evaluate | Phone the older adult and follow-up on the HIP-REP intervention | Assess results and evaluate | Phone the older adult and follow-up on the HIP-REP intervention |  |
| Week 12 | 10th | Evaluate | **Evaluate** | Evaluate | **Evaluate** |  |
| ADL; Activity of daily living; HIP-REP; Hip fracture REhabilitation Program; OTIPM; Occupational Therapy Intervention Process Model; PADL; Personal Activity of daily living; IADL; Instrumental Activity of daily living | | | | | | |
