# Supplementary material for: HIP Fracture REhabilitation Program for older adults with hip fracture (HIP-REP) based on activity of daily living: a feasibility study
Source: BMC Geriatr. 2022 Apr 27;22:370. doi: 10.1186/s12877-022-03039-x (PMC9044869; doi:10.1186/s12877-022-03039-x)
Supplement: Supplementary file 2 — Additional file 2: S2. Primary outcomes in research progression criteria to inform the definitive randomized controlled trial. [file 12877_2022_3039_MOESM2_ESM.docx]

| **S2** Primary outcomes in research progression criteria to inform the definitive randomized controlled trial | | |
| --- | --- | --- |
| Research progression rate | | Evaluation |
| Participant recruitment rate (*n*/month) | 4.5 | Green (go) |
| Completion of the outcome measures  Assessment duration including screening (min-max),  and both HCPs and older adults found the duration to  long | < 90 min. | Amber (amend) |
| Participant retention (*n*/%) | 13 (72.2) | Green (go) |
| Adherence to intervention sessions  Participants who completed the follow up (*n*/ %) | 13 (72.2) | Green (go) |
| Adverse events  Minor adverse events (*n*)  Serious events (*n*)  Participants discontinuing the study (*n*) | 1  0  0 | Green (go) |
| The above progression criteria were based on a traffic light system of green (go), amber (amend) and red (stop) (40). | | |
